# The anxiety associated with COVID-19, general health, spiritual health, and job satisfaction in healthcare providers: a cross-sectional study

**DOI:** 10.1186/s40359-023-01283-3

**Published:** 2023-08-23

**Authors:** Daem Roshani, Keivan Saboni, Mohiadin Amjadian

**Affiliations:** 1 Health Metrics and Evaluation Research Center, Kurdistan University of Medical Sciences, Sanandaj, Iran; 2 Department of Surgery, School of Medicine, Kurdistan Medical Sciences University, Sanandaj, Iran; 3 English Language Department, School of Medicine, Kurdistan Medical Sciences University, Sanandaj, Iran

**Keywords:** COVID-19, Anxiety, General health, Job satisfaction, Spiritual health, Healthcare worker

## Abstract

**Background:**

Previous research has shown the impact of pandemic communicable diseases on the mental health of healthcare providers. This study examined the relationship between general health, spiritual health, anxiety associated with COVID-19, job satisfaction, and the mediating role of sex in healthcare providers in Iran in 2021.

**Methods:**

This was a descriptive-analytical and cross-sectional study performed on 163 healthcare providers of which 71.8% were female and 28.2% were male with an age range between 21 and 58 years, an average work experience of 9.5 years, and working as nurses, doctors, laboratory technicians, etc. in some hospitals in Sanandaj. Data were collected using COVID-19 Anxiety, General Health, job satisfaction, and Spiritual Health questionnaires. Then, the data were analyzed using SPSS-22 software, regression test, and path analysis.

**Results:**

Although women averaged lower levels of general health, job satisfaction, and anxiety associated with COVID-19, and higher scores in spiritual health than men, none of these differences were statistically significant, and sex didn’t play a significant role here. Also, general health and spiritual health could significantly predict 17.1% of the variance in job satisfaction in the path analysis. However, sex and Covid-19 anxiety could not significantly predict this variable.

**Conclusion:**

The results showed that there was not a significant difference between the male and female workers’ general health when facing such pandemics. However, we may prepare interventions to promote their general and spiritual health and to promote healthcare providers’ job satisfaction during such pandemics.

## Background

Coronavirus disease (COVID-19) is a highly infectious respiratory disease caused by severe acute respiratory syndrome-coronavirus-2 (SARS-Cov-2) [1]. Research conducted in China during the COVID-19 pandemic found lower levels of mental health alongside a higher rate of anxiety, depression, and alcohol use than usual ratio [2], also one-third of people have reported lower mental well-being [3]. The pandemic situation has also affected people’s lives. Individuals experienced feelings of fear, and anxiety during the pandemic [4]. In humans, fear can reach high levels and arouse further negative emotions [5, 6] increasing the risk of physical and psychopathological problems [7]. Moreover, all reviews agree that the pandemic has increased mental health problems globally, with generalized fear as the central feature of its psychological impact [8, 9].

The extraordinary burden created by the epidemic on every nation’s healthcare structure has presented numerous trials to the nurses and other medical workers, which may cause disturbances to their mental health and overall work performance by creating depressive, anxious, and stressful situations [10, 11]. So, studies investigating mental health problems and the associated factors among front-line health professionals during an outbreak are very essential to planning the strategies needed to combat emotional distress [12]. For example, the results of Fathi et al. showed that among the psychological disorders, paranoia and general health could predict the anxiety associated with COVID-19 in students after the first pick of the epidemic [13]. Also, Por Kenari et al. showed that by adjusting the underlying and intervening variables, among the dimensions of job satisfaction, the colleague (p > 0.049) and the work environment (p > 0.043) were related to their general health [14]. The study of Khandan et al. also showed a significant relationship between job satisfaction and general health [15], and the study of Khamseh et al. showed that occupational and environmental stressors in healthcare providers increased the likelihood of emotional reactions such as depression, anxiety, and stress [16]. Moreover, a study in a Japanese hospital in 2013 on anxiety, stress, and depression in working female nurses indicated that these factors could have devastating psychological as well as physical effects on them [17]. Also, Sakr et al. study on anxiety in healthcare providers during COVID-19 Pandemic in Lebanon showed that the majority (83%) had minimal to mild anxiety, whereas the rest had moderate to high anxiety levels [18]. Finally, the study by Abid et al. revealed a significant predictive link between fear of COVID-19 and depression, anxiety, and stress in nurses in Pakistan [19].

On the other hand, Hatamipour et al. showed that there was a significant and negative relationship between spiritual health and anxiety in nursing students, so those with high spiritual health had milder anxiety [20]. The results of another study showed that the higher the level of spiritual health in nurses, the lower the anxiety associated with Covid 19 disease and vice versa [21]. Moreover, Khorami Markani et al. showed a significant positive and direct correlation between spiritual health and job satisfaction in hospital staff and considered the use of religious and spiritual teachings to be effective in increasing their job satisfaction [22]. Finally, Coppola et al. also showed that spirituality and religious practices were protective factors connected not only with psychological and mental but also physical health, and women perceived lower mental health than men during the COVID-19 pandemic in Italy [23].

All over the world, various studies have explored the effects of Covid-19, but unfortunately, only a few have revealed the emotional problems arising due to the fear of COVID-19 in healthcare providers [24]. So, the objective of this study was to investigate the relationship between general health, spiritual health, Covid − 19 anxiety, and sex with job satisfaction, among frontline healthcare providers in some hospitals in Sanandaj during the COVID-19 pandemic. Therefore, the hypothesis was as follows: there was a relationship between general health, spiritual health, Covid − 19 anxiety, and sex with job satisfaction among frontline healthcare providers in Sanandaj hospitals during the COVID-19 pandemic.

## Methods

### Participants

This descriptive-analytical and cross-sectional study was  performed on healthcare providers in some hospitals of Sanandaj in Iran in 2021. The participants were selected randomly, having the list of the healthcare providers in all wards with numbers and selecting the odd numbers from each ward list. Inclusion criteria included all healthcare providers working in different hospital wards between 18 and 60 years old during the last month of the COVID-19 pandemic. Exclusion criteria included those older than 60 years old and those who started working in the hospitals less than one month. The anxiety associated with COVID-19, general health, spiritual health, and job satisfaction were the variables studied and sex was supposed to have a role as a mediator.

### Data Collection

To collect the data, general health, spiritual health, job satisfaction, and anxiety associated with COVID-19 questionnaires were simultaneously presented to the participants online using Persian Press Online software in two different parts including demographic data such as the participants’ national code, sex, age and the ward and hospital name, and the questionnaires. Then, the scores of the participants in each questionnaire were calculated and entered into the analysis as a unit factor. To prevent any potential source of bias, a number was assigned to each participant’s national code to ensure the anonymity and confidentiality of their data, and the participants’ written consent was obtained first. The sample size was calculated to be 180 using Cochran’s statistical formula.

### Tools

#### Demographical information

The demographic information questionnaire included several questions about age, sex, hospital, and job type.

#### General Health

The General Health Questionnaire was designed by Goldberg and Hiller and localized in Iran by Taghavi ^28^. In the study of Taghavi, the reliability calculated by Cronbach’s alpha method for the whole questionnaire, somatic symptoms, anxiety and sleep disorder, social function, and depression symptoms were 0.76, 0.76, 0.86, 0.61, and 0.88 respectively. If the score obtained from each subscale was higher than 6 or more than 22 in the total score, it indicated that the person was unhealthy and ill. In other words, a higher score indicated lower and poorer health [25].

#### Spiritual health

The twenty-item Spiritual Health Questionnaire was developed by Polutzin and Ellison in 1982. The reliability of this questionnaire was calculated for the whole questionnaire, and its religious and existential dimensions were 0.83, 0.83, and 0.60, respectively using Cronbach’s alpha method [26]. Also, Safaee Rad et al. reported Cronbach’s alpha coefficient for the whole scale, and religious and existential components to be 0.90, 0,87, and 0.87, respectively [27].

#### Job satisfaction

Minnesota Job Satisfaction Questionnaire, developed by Weiss-Davis George England and Lafcastle in 1967 at Minnesota State University, is widely used to measure satisfaction in health-related occupations such as nursing, education, factories, manufacturing jobs, and Management. Scores were calculated in the range of 20 to 100. In the present study, Cronbach’s alpha coefficient for the whole scale was calculated to be 0.92 [28].

#### Measuring anxiety caused by COVID-19

To measure anxiety associated with COVID-19 in individuals, a questionnaire called COVID-19 Anxiety Scale was used. This questionnaire was prepared to measure anxiety caused by the outbreak of Coronavirus in Iran, and it has recently been validated by Alipour et al. The scale has 18 items that generally measure two components. items 1 to 9 assess psychological symptoms, and items 9 to 18 assess physical symptoms of anxiety. The validity of the questionnaire was evaluated by correlation-dependent validity using the Goldberg Standard General Health Questionnaire. The results indicated a significant correlation with the components of this questionnaire [29].

### Data analysis

The data were analyzed using a statistical package for the social sciences (SPSS) software version 26. The normality of variables was confirmed through the examination of skewness, kurtosis cures, and K-S test. Missing data were deleted from the analysis. Then OLS regression was used to investigate any relationships between the anxiety associated with COVID-19, general health, spiritual health, and job satisfaction. Given that the statistical distribution of all parameters was normal, correlation coefficients were calculated by the Pearson test. A theoretical model was then described in AMOS software for path analysis. Path analysis is a casual modeling approach to exploring the correlation within a defined network of variables. The comparative fit index(CFI), Tucker Lewis index (TLI), root mean square error of approximation(RMSEA), and the model chi-square (x^2^ ) were used to evaluate the overall model fitness. Acceptable model fit is detected when CFI and TLI values are $>0.90$, and RMSEA is $<0.10.$ Moreover, *R*^2^, the determination coefficient, was performed showing the variance percentage to establish the predictive power of the model. Indicators of each variable were fixed to 1.0 to create a metric scale. Further, all variables were fixed to 0 error, which is commensurate with OLS regression analysis path models. The significant level used was 0.05, meaning the test result was acceptable.

The initial model was proposed as follows:

#### Initial model


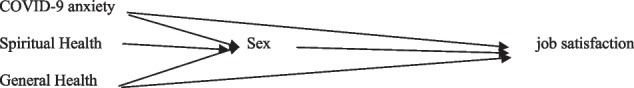


## Results

Demographic data indicated that 163 healthcare providers participated in the present study (about 9% left the study), of which 71.8% were female and 28.2% were male with an age range between 21 and 58 years, and an average of 33.7 years old. Their average work experience was 9.5 years, and they have been working as nurses, doctors, laboratory technicians, etc. 7.3% of participants had the least problems in general health, 30.1% had mild problems, 54.6% had moderate problems, 11.7% had severe problems. 33.1% of the participants had mild, 58.9% moderate, and 8% severe anxiety levels associated with COVID-19. Also, 14.7% of the participants had low, 50.9% had moderate, and 34.4% had high job satisfaction levels. Moreover, 2.5% of the participants had low, 72.4% had moderate, and 23.9% had high spiritual health scores.

Although women averaged lower general health, lower job satisfaction, lower anxiety associated with COVID-19, and higher spiritual health than men did, none of these differences were statistically significant. Therefore, the participants’ sex did not play a significant role in mediating the relationships among these variables (Table 1).

**Table 1:** Prediction of job satisfaction based on general health, spiritual health, gender, and anxiety associated with COVID-19

Predictive variables	B	SED	β	t	Sig.
**General Health**	0.256	0.072	0.269	3.583	0.000
**Anxiety**	− 0.067	0.085	− 0.058	− 0.792	0.429
**Spiritual Health**	0.394	0.106	0.274	3.706	0.000
**Sex**	0.041	0.109	0.027	0.374	0.709

Dependent variable: job satisfaction **[*P<*0.0005]**

### R Square = 0.171

Table 1 showed that general health and spiritual health were able to significantly predict participants’ job satisfaction in the regression analysis. Participants’ sex and anxiety associated with COVID-19 could not significantly predict this variable. General health and spiritual health were able to predict 17.1% of the variance in job satisfaction. The obtained model was generally significant (F = 13.969, *P*˂ 0.0005, *R*² = 0.171).

**Fig. 1:**
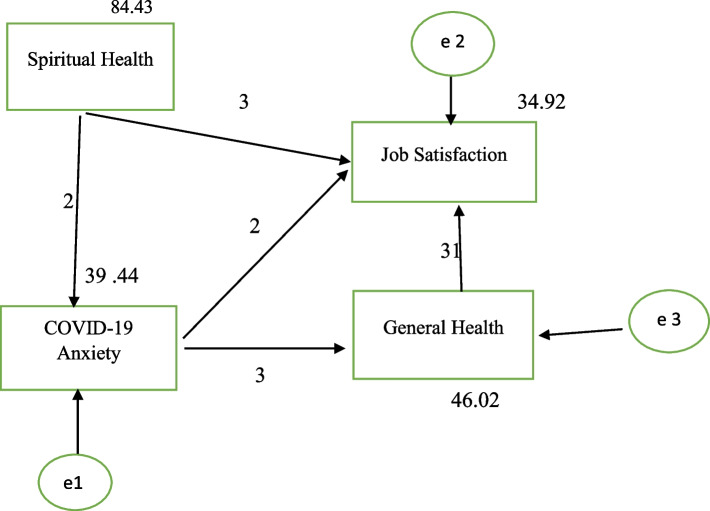
The final model path analysis

Finally, gender was not able to significantly predict job satisfaction and was excluded from the path analysis. Based on the significant correlation and linear regression results in the two stages, the following path analysis model was proposed by the researchers (Fig. 1). Based on this model, general health and spiritual health significantly predicted the level of job satisfaction. The latest approved and modified model in AMOS software was as follows (Fig. 1).

Since the Chi-square of the model was 1.885 [ Chi-square = 1.885, Sig. = 0.173], the model was approved. The values of the comparative indices were all close to one, indicating a good fit for the model (Table 2).

**Table 2:** Goodness of fit indexes for the revised model

Comparative indexes	CFI	NFI	RFI	IFI	TLI
**Values**	0.958	0.777	0.578	0.966	0.749

On the other hand, the value of PCLOSE was less than 0.05 and equal to 0.003 and the value of RMSEA was equal to 0.073 showing that the model was close to the acceptable model. Also, CMIN / DF = 1.885, which indicated the optimal model (Table 2).

## Discussion

This study aimed to determine the relationship between general health, spiritual health, and anxiety caused by COVID-19 disease with job satisfaction among the healthcare providers in Sanandaj hospitals. The results showed that 66.3% of participants have moderate to severe problems in their general health, 66.9% suffered from moderate to severe anxiety associated with COVID-19, 65.6% had moderate to low job satisfaction, and 74.9% had moderate to low spiritual health. Women had lower general health, less job satisfaction, lower anxiety, and higher spiritual health than men; however, none of these differences were statistically significant. This finding was consistent with the results of Khamseh et al. in which about 30% of nurses did not have good general health but there was no statistically significant difference between the two groups of men and women [16]. Also, the results were in line with Abid et al., which revealed a significant predictive link between fear of COVID-19 and depression, anxiety, and stress in nurses [19], but it was inconsistent with Kikuchi et al., in which the female nurses suffered from higher levels of depression, anxiety, and stress [17], and it was also inconsistent with Sakr et al. in which the majority of the participants had minimal to mild anxiety whereas a minority had moderate to severe anxiety levels [18].

The results also showed that general health and spiritual health were able to significantly predict the job satisfaction of the participants both directly, and indirectly through the anxiety caused by COVID-19 disease. On the other hand, sex did not significantly predict this variable. These findings were in line with the results of the study of Por Kenari et al., and Khandan et al. in which general health was significantly related to job satisfaction [14, 15]. Similarly, the results showed that spiritual health was a significant predictor of job satisfaction both directly, and indirectly through anxiety caused by COVID-19. These were consistent with the findings of Coppola et al. which showed that spirituality and religious practices are protective factors connected not only with psychological and mental but also physical health, and women had lower mental health than men [23], and they were also in line with Hatami Pour et al., Mohammad Zade Tabrizi et al., and Khorami Markani et al. [20–22]. However, in the study of Fathi et al., general health was able to predict the level of anxiety caused by Covid-19 disease after the first pick of the pandemic in students [13] which was inconsistent with the finding of this study. The low level of anxiety associated with COVID-19 and its insignificant relationship with general health might be related to the fact that the present study was performed when all healthcare providers had received the first dose of the COVID-19 vaccine in Iran. Therefore, there could be to be a relationship between the anxiety and fear caused by this disease and the vaccination among these healthcare providers.

Given the correlation between spiritual health and general health with job satisfaction in this study and also in several similar studies, it is very likely that general health and spiritual health can predict job satisfaction and anxiety associated with Covid-19. Therefore, to improve job satisfaction and reduce anxiety and fear of COVID-19 in healthcare providers, it is recommended to have a comprehensive stress management program for all healthcare providers, not to employ those with high stress and inexperienced in high-risk wards, to provide psychological counseling services to healthcare providers of hospitals, as well as to monitor and examine their physical and mental health regularly. In the comprehensive programs to increase general health and reduce anxiety associated with high-risk diseases such as COVID-19, holding workshops on interpersonal skills such as verbal, and auditory skills on how to communicate effectively with patients and also with colleagues, increasing hospital managers’ knowledge, using healthcare providers’ opinions in working decisions, and also paying attention to their welfare and personal problems seem to be very important.

One of the strengths of this study was that the sample included all healthcare providers including physicians, nurses, administrative, and para-clinical staff which made the study more comprehensive so that the results might be generalizable to the whole population in the hospitals.

The most important limitation of the present study was the limited number of samples, which was caused by the small number of hospitals in the study area. Also, the study was conducted in the fourth pick of the COVID-19 pandemic, therefore there was a severe limitation in face-to-face administrating the questionnaires. Ignoring background contexts and skills such as the participants’ jobs (doctor, nurse, etc…), age, and any previous psychological disorder was another limitation of this study. Moreover, the use of a self-administered questionnaire and the cross-sectional character of the research were other limitations of the present study; as a result, we were unable to draw a cause-and-effect relationship.

It is recommended to study the effects of interventional programs such as psychological and educational programs on the necessary skills to find how general and spiritual health might affect job satisfaction and anxiety caused by high-risk and communicable diseases like Covid-19 among healthcare providers.

## Conclusion

Healthcare providers experienced high levels of anxiety, and low general health and job satisfaction during the COVID-19 pandemic. The results of this study may help prepare appropriate future interventions and effective prevention programs to reduce their anxiety and promote their general health and job satisfaction during such pandemics.

## Data Availability

The datasets used and/or analyzed during the current study are available from the corresponding author on request.
